# Comparative Analysis of Phenology Algorithms of the Spring Barley Model in APSIM 7.9 and APSIM Next Generation: A Case Study for High Latitudes

**DOI:** 10.3390/plants10030443

**Published:** 2021-02-26

**Authors:** Uttam Kumar, Julien Morel, Göran Bergkvist, Taru Palosuo, Anne-Maj Gustavsson, Allan Peake, Hamish Brown, Mukhtar Ahmed, David Parsons

**Affiliations:** 1 Department of Agricultural Research for Northern Sweden, Swedish University of Agricultural Science, 90183 Umeå, Sweden; julien.morel@slu.se (J.M.); anne-maj.gustavsson@slu.se (A.-M.G.); mukhtar.ahmed@slu.se (M.A.); david.parsons@slu.se (D.P.); 2 Department of Crop Production and Ecology, Swedish University of Agricultural Science, 705007 Uppsala, Sweden; goran.bergkvist@slu.se; 3 Natural Resources Institute Finland (Luke), FI-00790 Helsinki, Finland; taru.palosuo@luke.fi; 4 CSIRO Agriculture and Food, Canberra 2601, Australia; allan.peake@csiro.au; 5 The New Zealand Institute for Plant and Food Research Limited, Private Bag 4704, Christchurch 8140, New Zealand; hamish.brown@plantandfood.co.nz

**Keywords:** phenology, barley, modelling, algorithms, APSIM next generation, APSIM classic, high latitudes

## Abstract

Phenology algorithms in crop growth models have inevitable systematic errors and uncertainties. In this study, the phenology simulation algorithms in APSIM classical (APSIM 7.9) and APSIM next generation (APSIM-NG) were compared for spring barley models at high latitudes. Phenological data of twelve spring barley varieties were used for the 2014–2018 cropping seasons from northern Sweden and Finland. A factorial-based calibration approach provided within APSIM-NG was performed to calibrate both models. The models have different mechanisms to simulate days to anthesis. The calibration was performed separately for days to anthesis and physiological maturity, and evaluations for the calibrations were done with independent datasets. The calibration performance for both growth stages of APSIM-NG was better compared to APSIM 7.9. However, in the evaluation, APSIM-NG showed an inclination to overestimate days to physiological maturity. The differences between the models are possibly due to slower thermal time accumulation mechanism, with higher cardinal temperatures in APSIM-NG. For a robust phenology prediction at high latitudes with APSIM-NG, more research on the conception of thermal time computation and implementation is suggested.

## 1. Introduction

Process-based crop models simulate dynamic and complex interactions between environment, genotype, and management factors. Various algorithms and parameters in crop models simulate different plant and soil processes on several interactions and linkages. The processes related to phenology, dry matter accumulation and partitioning, and soil hydrology and chemistry are simulated with the various algorithms and parameters. Model algorithms and parameters are still simplifications of real systems [1], which makes crop models contain unavoidable systematic errors.

Additionally, at the user level, the quality of input data [2] and the choice of model parameterization approach [3] create further uncertainties. Since crop models are being extensively applied to a wide range of agricultural research questions and hypothesis testing, such as assessments of climate change effects [4], decision making and planning [5], farmer advisory [6], crop–livestock systems [7,8], agronomic management [9], physiological mechanisms and traits [10,11], linking phenotype to genotype [12,13], and plant breeding [14,15], it is critical to find ways to reduce the uncertainties.

Most of the commonly and widely used crop models (e.g., DSSAT, APSIM, CROPSYST, EPIC, STICS, WOFOST, DAISY, ORYZA, GLAM, and INFOCROP) were constructed at least two decades ago using crop data obtained under controlled growing conditions and limited datasets [16]. Since the development of the models, improving their prediction accuracy has been the main emphasis [17,18] to better adapt them for new environments and future research questions. Suggestions for identifying and improving the prediction accuracy of various processes have been an important part of several recent crop model ensembles and inter-comparison studies [19,20,21,22,23].

More specifically, studies have highlighted that robust phenological submodels are crucial tools to improve the accuracy of crop models [24,25], given the importance of the timing of anthesis and crop duration in crop yield determination [26,27]. Such improvements have great importance and can increase confidence and reliability when simulating the response of plants, for example, to climate change [28] and temperature [24], to better plan agronomic activities to maximize crop yield while reducing the risks. Interestingly five different phenological models/modules (i.e., CERES-Rice, ORYZA2000, RCM, Beta Model, SIMRIW) were compared and reported to simulate varied plant responses to climate change and variability in different regions [29]. The variations were more evident when the temperature in the regions was above or below the optimum temperature, as defined for optimum growth in the models. In crop models, thermal time accumulation is sometimes implemented with piecewise non-linear response functions with temperature-limit values called cardinal temperatures. With different cardinal temperatures and response functions used to compute thermal time for the targeted developmental stages, ORYZA2000 and CERES-Rice simulated varied phenology [24].

Various crop growth processes implemented in crop models, such as dry matter accumulation and partitioning, leaf number, and leaf area dynamics, are strongly linked with phenological development [25,30]. Rötter et al. [31] found incongruities in the predictions of crop phenology and yield. The study compared and applied nine crop models at seven different locations in Northern and Central Europe and reported that models such as WOFOST and STICS were more accurate in simulating phenology; however, they were not necessarily the best predictors of the grain yields. This can be associated with the fact that a small error in phenology can have a large influence on the yield, especially if errors occur during sensitive growth stages, such as fertilization. Thus, to increase the overall accuracy of crop models, it is critical to better synchronize phenological simulations with other processes in the model.

APSIM is currently one of the most widely used crop and farming systems models globally [32]. With the increasing challenges and demand for agricultural modelling, the APSIM initiative and its predecessor The Agricultural Production Systems Research Unit (APSRU) have been constantly improving and building APSIM model components since its release in 1990, in order to improve its prediction accuracy and increase its applicability to a wider range of farming systems [33]. An example of this effort is the implementation of a new phenology simulating mechanism in the recently released APSIM next-generation (APSIM-NG) models compared to the one used in the classical versions of APSIM (APSIM 7.x).

Spring barley is ranked fourth in world cereal production and is particularly important in boreal regions with short growing seasons, such as at high latitudes [34]. Although there have been several studies on barley using APSIM and other crop models (e.g., [21,31]), achieving a robust calibration is still a challenging task when less detailed experimental datasets are available [3]. The availability of good datasets generated at high latitudes are particularly poor in terms of data on critical growth stages, which limits the robustness of calibration and makes models prone to having poor prediction accuracy. For the same reason, the strengths and weaknesses of the APSIM barley model still need to be critically assessed at high latitudes for further application as a research tool. Therefore, the data on days to anthesis and physiological maturity on recently bred barley varieties were used in the study to evaluate the phenology simulation mechanisms of APSIM 7.9 and APSIM-NG. Focusing on one aspect of the simulating mechanism (in this case, phenology) allows for a more systematic assessment of the contributing factors to the uncertainty and the targets for improvement [31]. The relevance of assessing the models using current varieties, which were developed considering the short and risky cropping seasons experienced at high latitudes, particularly for phenology-simulating mechanisms, is significant and applicable in terms of their potential use in the region [35,36].

This study aimed to evaluate the phenological algorithms of the APSIM classical version 7.9 (hereafter APSIM 7.9) and the APSIM-NG (version APSIM2019.11.27.4417) models of barley using experimental data for recently developed barley varieties from high latitudes (north and central Sweden and central Finland). The specific objectives of the study were to 1) calibrate and evaluate phenology algorithms of APSIM 7.9 and APSIM-NG barley models using a smaller and larger dataset on days to anthesis and physiological maturity, and 2) identify potential sources of error in the algorithms and suggest means for future improvement.

## 2. Results

### 2.1. Crop Phenology Overview

**Figure 1:**
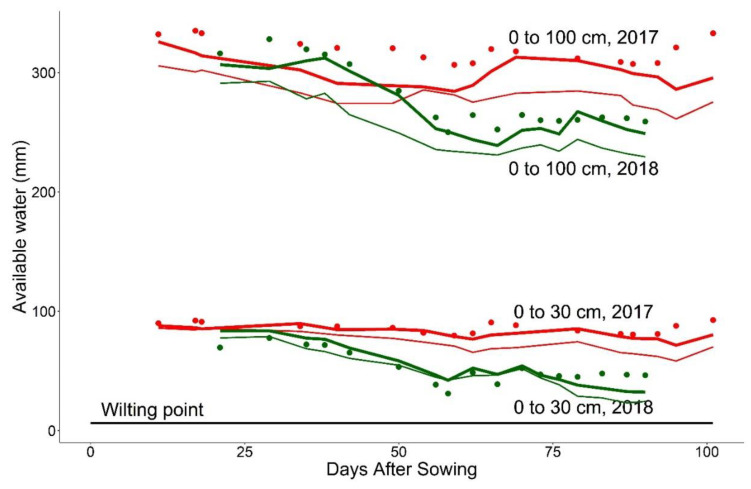
Soil moisture dynamics of 1-m soil profile with ten layers of equal depths during the 2017–2018 cropping seasons. The upper part of the figure represents the whole profile (0 to 100 cm) and the lower represents the topsoil layers (0–30 cm). Volumetric water content (%) (VWC) is the volume of water per cubic unit of soil, shown as available water (mm). The points represent measured data and continuous lines represent simulated data. Thick lines are for APSIM 7.9, and thin lines are for APSIM-NG. Wilting point is defined as 6.39% of VWC based on the data in Table S1. 2017: Red lines and points, 2018: green lines and points.

All the varieties, on average, needed more days to reach physiological maturity in the colder cropping season in 2017 than in the warm and dry cropping season of 2018 (Table 1). On average, across the locations, varieties reached physiological maturity 33 days earlier in 2018 than 2017. At Röbäcksdalen in 2017, the variation in days to anthesis among the varieties was less than the variation in days to physiological maturity. Compared with 2017, the variation and duration of days to anthesis and physiological maturity were lower in 2018. The soil moisture and phenology data at Röbäcksdalen suggest that barley development was rapid as the warm and dry conditions intensified 40 days after sowing in 2018 (Figure 1 and Table 1. The years 2014 and 2018 were similar with respect to days to physiological maturity, whereas the years 2015 and 2016 were intermediate in terms of maturity in 2017 and 2018. On average, barley matured latest at Ås and earliest at Offer and Ruukki.

### 2.2. Calibration and Evaluation of APSIM 7.9

For *Calibration 1_AN*, RMSE was between 0 and 1.3 d, depending on the variety (Table 2). These RMSEs were achieved with *photop_sens* between 0 and 1, *vern_sens*, 0 and 0.5; *tt_end_of_juvenile*, 200 or 300; *tt_floral_initiation*, 300 or 320. For *Calibration 2_PM*, when the object variable was days to physiological maturity, the RMSE range among varieties was between 2.2 and 8.6 d, a wider range than for *Calibration 1_AN*. The wider range of RMSE was because of the different combinations of the parameters, a more variable *photop_sens* (0–6); the same range of *vern_sens*, the same range of *tt_end_of_juvenile* but with a few entries of 250, depending on the variety, same range of *tt_floral_initiation,* and *tt_start_grain_fill* between 500 and 625. It should be noted that different combinations of the parameters resulted in the same RMSEs, particularly for *Calibration 1_AN*, which is a common phenomenon, termed equifinality, when a large number of combinations are tested [37].

For *Calibration 3_PM,* RMSE for different varieties was between 7.5 and 11 days (Table 2). Although the dataset for this calibration was larger than for *Calibration 2_PM*, the range of RMSE was broader. Compared to the previous two calibrations, here, *photop_sens* was between 0 and 3; *vern_sens,* 0, *tt_end_of_juvenile*, 250–450 with more higher values of 450; *tt_floral_initiation,* 240–320, and *tt_start_grain_fill*, 300 or 450.

Based on the combination of parameters that resulted in the best possible RMSEs in the above calibrations, it can be inferred that the combinations were different because of the target of the growth stage and the size of the dataset used for calibration.

**Figure 2:**
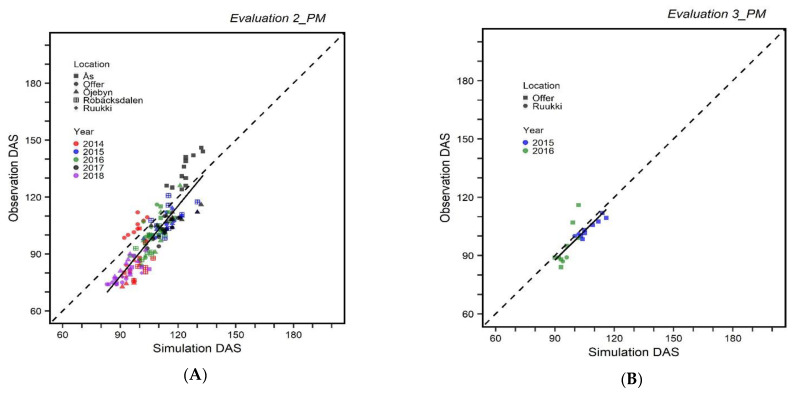
Evaluation of APSIM 7.9 barley model. (**A**) *Evaluation 2_PM* represents the evaluation with the data from 22 environments; (**B**) *Evaluation 3_PM* represents evaluation with the data from 4 environments. Each point represents simulation and observed data for one variety. Observed DAS and Simulation DAS are observed and simulated days after sowing outputs, respectively.

Parameters from *Calibration 2_PM* in the *Evaluation 2_PM* overestimated the days to maturity particularly for environments of early maturity and underestimated for late maturing environments (Figure 1A). However, in the *Evaluation 3_PM* for the parameters calibrated under *Calibration 3_PM*, the performance of the model was better, with lower RMSE terms and better *r*^2^ (Figure 2B and Table 3).

### 2.3. Calibration and Evaluation of APSIM-NG

For *Calibration 1_AN,* APSIM-NG showed very good calibration performance (RMSE zero days) for all varieties (Table 4). This RMSE was achieved with the following parameter values: *PpSensitivity,* zero, *VrnSensitivity*, 0.5–1.5; *BasePhyll,* 55 or 60; *MinimumLeafNumber,* 6; *EarlyReproductivePpSensitivity,* zero.

Similarly, for *Calibration 2_PM,* RMSE was zero days, indicating a better performance for APSIM-NG than APSIM 7.9 for the same calibration using the same dataset. This RMSE was achieved with the following parameter values: *PpSensitivity* equal to zero, *VrnSensitivity*, 0.5–1.5; *BasePhyll,* 50–60; *MinimumLeafNumber*, 5–6.5; *GrainFill,* 450–625; and *EarlyReproductivePpSensitivity,* zero. Similar to APSIM 7.9 different combinations of the parameters resulted in the same RMSEs under *Calibration 1_AN*. Therefore, the parameter combinations in Table 4 were selected with the same process as for APSIM 7.9.

For *Calibration 3_PM,* RMSE was between 4.6 and 7.5 days, depending on the variety (Table 4). Compared to the previous two calibrations, here the *PpSensitivity* was 0; there was a slightly wider range of *VrnSensitivity* (0–3) and *BasePhyllo* (50–65); the *MinimumLeafNumber* was 5–6.5, and the values for *GrainFill* were lower and narrower, 400–575; and *EarlyReproductivePpSensitivity* was 0.

As with APSIM 7.9, the best combination of the selected parameters for APSIM-NG was different depending on the target of the growth stage and the size of the dataset used for calibration.

*Evaluation 2_PM* showed that days to physiological maturity was consistently overestimated (Figure 2A). Twenty evaluation simulations did not reach physiological maturity and stopped at the end date of 30 November. The days after sowing of those incomplete simulations, together with the observed data, are presented in Figure 2A. Based on the completed simulations, the intercept was closer to zero compared to the same evaluation of APSIM 7.9 (Table 3). The consistent overestimation for the completed simulations resulted in a higher RMSE than for APSIM 7.9. However, with similar RMSE_sys_, and higher RMSE_nos_, the overall correlation (*r*^2^) was higher.

**Figure 3:**
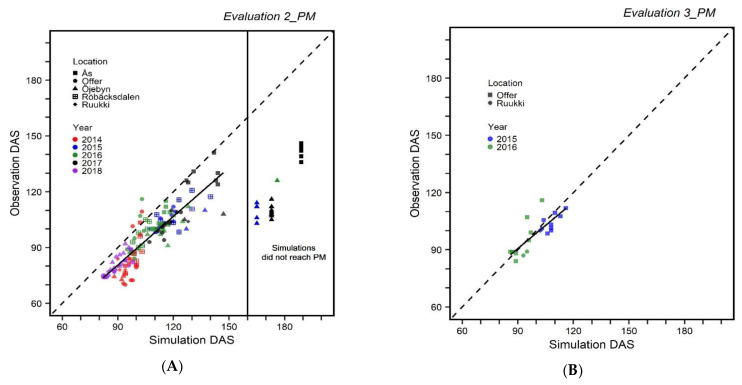
Evaluation of APSIM-NG barley model. (**A**) *Evaluation 2_PM* represents the evaluation with the data from 22 environments; (**B**) *Evaluation 3_PM* represents evaluation with the data from 4 environments. Each point represents simulation and observed data for one variety. Simulaitions that did not reach maturity were not included in the regression line.

When the APSIM-NG calibration was performed with a larger dataset than in the evaluation (*Evaluation 3_PM)* RMSE was still higher; however, RMSE_sys_ and RMSE_nos_ were lower compared with the same evaluation and dataset for APSIM 7.9. Intercept and slope showed the inclination of overestimation; however, this was less obvious than for *Evaluation 2_PM*. The overall relationship (*r*^2^) of simulated values with the observed ones was lower than for APSIM 7.9 (Figure 3B and Table 3). Similar to APSIM 7.9, RMSE, RMSE_sys,_ and RMSE_nos_ under *Evaluation 3_PM* were lower than for *Evaluation 2_PM*.

## 3. Discussion

### 3.1. Performance of Phenology Simulation Algorithms in the Calibration and Evaluation of APSIM 7.9 and APSIM-NG

Although the calibration of the parameters for simulation of days to physiological maturity for APSIM-NG was better when based on the RMSE, in both evaluations, slopes < 1 suggested that APSIM-NG with these calibrations was inclined to overestimate the days to physiological maturity, particularly for later maturing varieties. This was affirmed by the consistent overestimation of days to physiological maturity with higher RMSE, and a more variable range of *r*^2^ for APSIM-NG (Table 3). In contrast, APSIM 7.9 overestimated the duration of the varieties when they matured earlier and underestimated them when they matured later. This indicated that the thermal time accumulation for different growth stages in APSIM-NG, determined by the cardinal temperatures applied, might be more sensitive to colder days in the late crop-maturing phase.

Similarly, for the calibration of anthesis, a lower RMSE for APSIM-NG than for APSIM 7.9 indicated that APSIM-NG’s phenology algorithm and the parameters for simulating days to anthesis were more adjustable to capture the responses of varieties to warm and dry (2018) and wet and cold (2017) cropping seasons. However, neither of the models was evaluated for days to anthesis due to lack of data.

### 3.2. Differences between APSIM 7.9 and APSIM-NG Algorithms for Simulating Phenology

For broader reliability of the model prediction, a robust calibration is indispensable for building confidence when predicting the targeted variables in different environments [38,39,40,41]. In this study, three calibrations with the targets of different growth stages, using different data sizes, provided a more robust scrutinization of phenology-regulating parameters. The parameters in APSIM-NG simulated days to physiological maturity less accurately in the evaluations compared with APSIM 7.9. As mentioned above, the reasons for this could be linked with cardinal temperatures (which were not calibrated) and their usage in the thermal time computations. In addition, the implementation of vernalization and photoperiod effects in the algorithms could be equally important.

The implementation of Kirby’s Framework [42,43] in APSIM-NG to capture the effects of vernalisation and photoperiod in sync with the Haun stage might provide better synchronicity and continuity in processes such as leaf emergence, floral primordia initiation, and anthesis, which could be the reason for the better calibration of APSIM-NG.

Thermal time computation in both APSIM-NG and APSIM7.9 models is achieved with the same approach (mean of maximum and minimum temperature during the day, and from which the base temperature is subtracted), but with different cardinal temperatures. Cardinal temperatures in both models, like other crop models, are crop-specific parameters. The new phenology simulation mechanism in APSIM-NG utilizes higher cardinal temperatures compared to APSIM 7.9. These parameters were not calibrated in this study, for two reasons: (1) the complete dataset on both growth stages was only for two seasons and one location, which we think were not enough to manipulate crop-specific parameters; (2) we aimed to test the phenology algorithms and parameters in their original form.

Apart from the usage of different cardinal temperatures, another difference that can lead to different responses in these models is employing the crown temperature for the computation of thermal time. In APSIM 7.9, air temperature is first converted into crown temperature, and then the thermal time computation is made. However, in APSIM-NG, instead of using crown temperature for the computations, higher base temperature (Tb), optimum temperature (Topt), and maximum temperature (Tmax) are used as proxies for crown temperature. In APSIM-NG, the response function of thermal time computation has two slopes, with three air cardinal temperatures: Tb = 4 °C, Topt = 26 °C, (a positive slope from Tb to Topt) and maximum Tm = 37 °C, with a negative slope from Topt to Tm that results in 0, 22 and 0 °Cd thermal time for the respective cardinal temperatures. In APSIM 7.9, the air cardinal temperatures are, Tb = 0 °C, Topt = 26 °C and Tm = 34 °C, with the corresponding thermal times as 0 °Cd, 26 °Cd, and 0 °Cd, with the same response function as in APSIM-NG. This suggests that APSIM-NG has a slower thermal time accumulation mechanism compared with APSIM 7.9, which resulted in the overestimation of later maturing varieties when the temperature was relatively lower in the later crop maturing phase. Due to the slower thermal time accumulation, twenty simulations did not complete physiological maturity. For APSIM 7.9, the accumulation was faster, hence it underestimated maturity for some of the late-maturing varieties.

Similar to other models, such as DSSAT, STICS, GLAM, WOFOST, DAISY [44,45,46,47,48], the linear response functions in APSIM-NG and APSIM 7.9 that guide the developmental rate based on the cardinal temperatures assume a rapid increase in the developmental rate above the optimum temperature compared to the rate between base and optimum temperatures. Such an incorporation can lead to poor predictions and systematic errors [49,50]. In this study, the average temperature was below the optimum temperature during the studied cropping seasons, and the prediction of days to maturity in the evaluations was still not good, particularly for APSIM-NG. This indicated that the rate of thermal time accumulation with the default optimum temperature or the response function was not the primary reason in the models for errors in simulating phenology.

The average temperature at the studied location was below 4 °C (Tb in APSIM-NG) for many days during the crop maturation phase. Earlier studies have reported that when the cardinal temperature at the study locations falls outside of the range of temperature used for algorithm development, prediction discrepancies can be observed [24,51]. Thus, it is possible that the higher Tb in APSIM-NG and, most likely, the computation of thermal time without considering the crown temperature, could be the primary reason for having a worse performance than APSIM 7.9 in some aspects in the evaluations.

The two different phenology simulation mechanisms in both versions of APSIM essentially make them two completely different models. Models with relatively simpler phenological simulating mechanisms, accounting for the effects of temperature and daylength (e.g., WOFOST, and FASSET), to more complex mechanisms, accounting for the effects of temperature, photoperiod, and vernalisation (e.g., DAISY and HERMES), have been reported to simulate variable phenology [20,29]. Due to such responses, much work has been conducted recently to improve phenology submodules by altering the cardinal temperatures, particularly at higher temperatures [52,53]. The current study is in line with other findings [24,51], which suggest that caution is needed in the use of cardinal temperatures, particularly when a model is to be used in a new environment where the temperatures may fall outside the range for which the phenological model was developed. This consideration is critical at high latitudes, where the night temperature during seedling and maturity stages can be low, as observed in the current study, where APSIM-NG, with a higher base temperature, took more days to simulate the days to physiological maturity than APSIM 7.9. However, the broader implications of different cardinal temperatures in the models can be assessed when they are calibrated and evaluated with comprehensive datasets. Therefore, further studies are recommended in this direction.

### 3.3. Equifinality and Selection of the Parameters and Their Influence On Phenology

The combinations of the best parameters for the varieties varied under each calibration. The different combinations are not surprising, since the targeted set of parameters that was calibrated under Calibration 1_AN was different than under *Calibration 2_PM* and *Calibration 3_PM.* Besides the different parameters, the dataset for each calibration was different. For *Calibration 1_AN*, data were used only on days to anthesis from two environments. For *Calibration 2_PM* and *Calibration 3_PM,* the data were on days to physiological maturity for two and twenty environments, respectively.

When several parameters regulate a mechanism, different combinations of the parameter values can result in the same solution, termed Equifinality [37]. In this study, the phenomenon was also observed. For example, a thermal type parameter in APSIM 7.9, tt_end_of Juvenile, has a potential range between 300 and 450 for Alvari (Table S3) in the quest to find the best solution. The parameter tt_end_of Juvenile and others that control phenological development do not stand alone in terms of simulating days to anthesis. Their role is coupled with the other parameters, i.e., tt_floral_initiation, photop_sens, and vern_sens. Therefore, specific combinations of the different parameters can result in the same output, and hence the same RMSE. However, our hypothesis for the equifinality in this study is that several parameters that were used to calibrate days to anthesis using the smaller dataset resulted in the same solution. The data on days to anthesis for the varieties were available only for two seasons, which, in mathematical terms, has two solutions for one growth stage for each variety, and with more than 14,560 (in the case of APSIM 7.9) parameter combinations, it was very likely that several combinations would achieve those two solutions. If the data on anthesis and earlier growth stages were available for more years and locations, then the number of best parameter combinations might be lower.

The selection of the best set of parameters based on high frequency corroborates the fact that their relevance for regulating phenology is higher, and they hence have a greater chance to more accurately simulate days to anthesis under several environments (years and locations) than others. Although the parameters regulating the growth stages before anthesis were calibrated on arbitrary assumptions due to lack of data for those stages, their sequential dependency on simulating days to anthesis makes them useful for the cases when the simulation target is to achieve accurate days to anthesis.

For calibration of days to anthesis under *Calibration 1_AN*, the parameter *PpSesnitivity* in APSIM-NG was more stable, with only one value across the varieties. This indicated that the parameter was more stable in the study locations. However, *PpSesnitivity*’s synonym parameter *photop_sens* in APSIM 7.9 was relatively more variable. Contrastingly, *VrnSensitivity* in APSIM-NG was more variable compared to *vern_sens* in APSIM 7.9. The parameter *MinimumLeafNumber,* regulating the leaf number in APSIM-NG, had only one value for all varieties: six leaves. Since *VrnSensitivity* changes with Huan stage (or leaf number) and given the dependence of leaf number on *BasePhylo*, a stable leaf number for the varieties was the result of the variable *BasePhylo* in combination with variable *VrnSensitivity*.

For the calibration of days to physiological maturity of the varieties under *Calibration 2_PM* and *Calibration 3_PM*, parameter combinations and variability were different within each calibration and with *Calibration 1_AN* for both models (Table 2 and Table 4). For *Calibration 2_PM* with the smaller dataset, on days to physiological maturity, *PpSensitivity* was the same, and *VrnSensitivity* had the same variability but had different values for the varieties compared with *Calibration 1_AN*. *BasePhylo* and *MinimumLeafNumber* became more variable. However, for most of the varieties, the algorithm selected one of the lower values for these parameters, which was used for the calibration. For *GrainFill,* these were mid-range values. With the larger dataset on days to physiological maturity under *Calibration 3_PM,* the combinations of the parameter changed again compared to the other two calibrations. Notable variations were observed with *VrnSensitivity, BasePhylo,* and *GrainFill*. This suggested that the large variations in the parameter *VrnSensitivity, BasePhylo, MinimumLeafNumber,* and *GrainFill* were more influential in regulating the duration of growth stages than *PpSensitivity* and *EarlyReproductivePpSensitivity*, which were unchanged.

Similarly, the combinations and variations in the parameters of APSIM 7.9 with different datasets suggested that all calibrated parameters were influential in regulating the duration of the growth stages. Contrastingly, a larger variability of *photop_sens* suggested that it was more influential in APSIM 7.9 than its synonymous parameter *PpSensitivity* in APSIM-NG. It was evident in both models that combinations of and variations in the best parameters changed under the calibrations with different sized datasets.

The responses of the parameters in the evaluations suggested that the error terms (RMSE, RMSEsys, and RMSEnos) were lower when they were calibrated with larger datasets compared to their calibration with smaller datasets. However, by assessing both models’ performance based on the error terms and *r*^2^ across the evaluations, it was difficult to point out which model was better.

### 3.4. Further Development of APSIM Barley Models for Northern Regions

In most crop models, thermal time accumulation is adjusted by simple functions to incorporate the effects of vernalisation and photoperiod. Such adjustments are usually linear, which makes the models rigid (biased) to predict accurate phenology at the locations and conditions where the linearity does not hold [24,51]. Palosuo et al. [20] reported that models that simulate crop phenology as a function of temperature, photoperiod, and vernalisation (e.g., DAISY and DSSAT) simulated wheat phenology better than the models that simulated crop phenology as a function of temperature and photoperiod only (e.g., WOFOST and FASSET) for northwestern, central and southeastern Europe. While comparing the same models as in [20], Rötter et al. [31] observed that phenology prediction discrepancies were linked with the temperature and photoperiod factors in barley models for northern and central Europe. To ease such linear rigidity, APSIM 7.9 models were incorporated with broken linear functions to accommodate the responses to temperature and photoperiod [54], which could be the reason it performed better with a large dataset in the evaluation in this study. Vernalisation accumulation is dependent on the temperature ranges during the cropping season. The duration to satisfying the vernalisation requirements could be different if the base temperature is different in the model, which was the case in the two models, and thus a different response was observed in this study simulating days to anthesis using the same dataset. Spring barley varieties, which are typically assumed to have no vernalisation requirement, were used in this study. By calibrating the vernalisation parameters, we did not automatically assume that there was no vernalisation effect, like in some some studies [55], and unlike others, such as Rötter et al. [31]. This is because although breeding programs have selected for the no vernalisation requirement, it is not completely eliminated. The magnitude of vernalisation requirements would be different to winter barley varieties using winter barley APSIM models. In both versions of APSIM, a three-hourly interpolation of maximum and minimum temperature is computed from the daily maximum and minimum temperature based on the method described [56]. The mean of such interpolation could be the same, irrespective of the different temperature amplitudes [57]. The three-hour estimate has an advantage, particularly at high latitudes where the effect of maximum temperature is greater than the minimum temperature during the spring cropping season due to long days. To further improve the prediction accuracy of phenology at higher latitudes in APSIM, and particularly APSIM-NG, addressing the temperature amplitude can be a target.

As previously mentioned, the excessive rain and low temperature during the crop-maturing phase in 2017 caused barley varieties to lodge or not to mature, whereas, in the hot and dry 2018 cropping season, varieties developed rapidly and produced the poorest yield since the 1950s [58]. By capturing such uneven climates at high latitudes, using non-linear functions could further increase the prediction capacity of the phenology algorithms [57]. Unlike crown temperature usage in APSIM 7.9, APSIM-NG does not directly use this for thermal time computation; instead, higher cardinal temperatures are considered, which could be another research area to reconsider, besides base temperature, to increase the robustness of APSIM-NG at high latitudes.

## 4. Materials and Methods

### 4.1. Location and Agronomic Management

**Figure 4:**
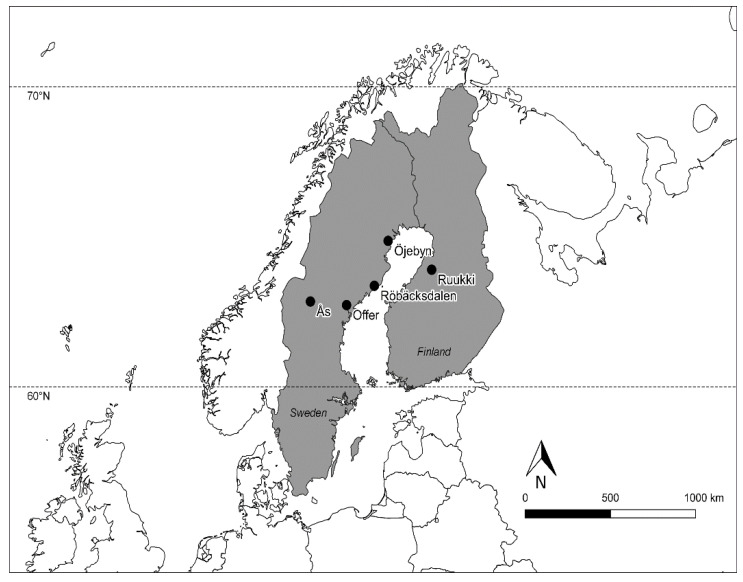
Study locations in northern Sweden and Finland.

Crop growth and phenological data for twelve spring barley varieties were collected or acquired from on-going official variety trials for four locations in northern Sweden, and one in northern Finland, for five cropping seasons (2014–2018) (Table 1). The varieties are modern and bred for short growing seasons at high latitudes. The varieties include both two-row and six-row barley. The locations for the study in northern Sweden were: Röbäcksdalen (63.80 °N and 20.18 °E), Öjebyn (lat. 65.34 °N and 21.39 °E), Offer (63.11 °N and 17.74 °E) and Ås (63.24 °N and 14.56 °E), and, in Finland, the location was Ruukki (lat. 64.36 °N and 25.22 °E) (Figure 4). The sowing dates and agronomic practices for five seasons at the study locations are provided in Table 1.

### 4.2. Phenological Data Collection

Frequent phenological developmental monitoring was conducted at Röbäcksdalen to obtain the precise days to 50% anthesis, and Zadoks stage 65 [59], (hereafter, days to anthesis) of twelve barley varieties for two cropping seasons (2017 and 2018) (Table 1). Yellow to brownish stamens in the panicles were observed as indicators of anthesis. Ten random panicles were chosen and dissected for the stamens from the barley plots in two replications. For the days to maturity (Zadoks stage 90), plant samples were collected from a 0.5 m^2^ quadrat from the middle rows of the plots. At each sampling date, at least 1 m of space was left while taking the samples. Plant samples were threshed and clean grains were obtained for recording fresh weight, and subsequently dried at 65 °C until there was no weight change. Using the date of sampling and dry matter content in the grains, a linear or 2nd-order regression was used to estimate the date of 70% dry matter in the grains to mark the loss of grain color of the peduncle and, hence, end of grain filling [60], which was recorded as physiological maturity for the crop. This measurement is in accordance with APSIM’s identification of the stages, i.e., end of grain filling and physiological maturity for barley.

The data for the varieties were complete for both phenological stages for the 2017 and 2018 cropping seasons at Röbäcksdalen. For other seasons and locations, the data were only for physiological maturity. Since not all twelve varieties were sown in every season and location, as they were in 2017 and 2018 at Röbäcksdalen, the data on the available varieties that were used for model calibration and evaluation are presented in Table 1. Missing values indicate that the variety was not grown or the measurement was not performed on time to compute the maturity date.

### 4.3. Soil Characteristics

Soil moisture (volumetric water content, %) was measured at Röbäcksdalen using Diviner2000 (Sentek technologies, Australia) from 11 to 138 days after sowing (DAS) in 2017, and 21 to 90 DAS in 2018, with an interval of 3–15 days, depending on the canopy development (Figure 1). The measurements were less frequent during early growth stages and more frequent during and after the closing of the canopy. In 2017 and 2018, two and one access tubes, respectively, were installed in each barley plot. There was no chronic or long water stress period during any of the cropping seasons, neither in the measurements nor in simulated data. Field capacity, saturation, wilting point and the pH of the soil were measured at Röbacksdalen, Offer, Ås, and Ruukki and used in the APSIM simulations (Table S1). The Swedish soil data are taken from existing studies [61,62]. The soils at all study sites are classified as Podzols (FAO). The Röbäcksdalen soil has a clayey silt loam topsoil [63], the Offer soil has a silty clay loam topsoil, the Ås soil has gravelly loam topsoil, the Öjebyn soil has a sandy topsoil [64] and the Ruukki soil has a sandy mull topsoil [65]. Figure 1 shows the simulation of the models with the input data for only one location (Röbäcksdalen) and two years. The results provided a base to assume that both models can simulate the patterns of soil water dynamics at the study locations.

### 4.4. Climate

**Figure 5:**
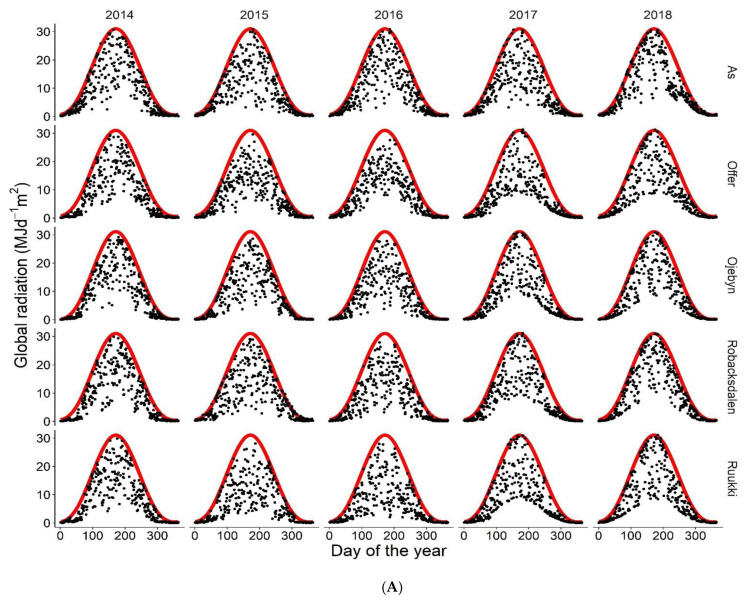

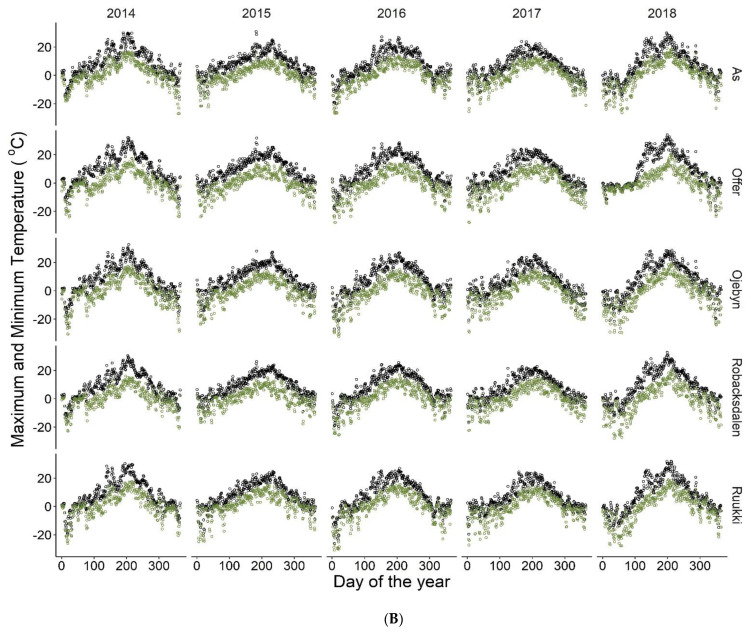

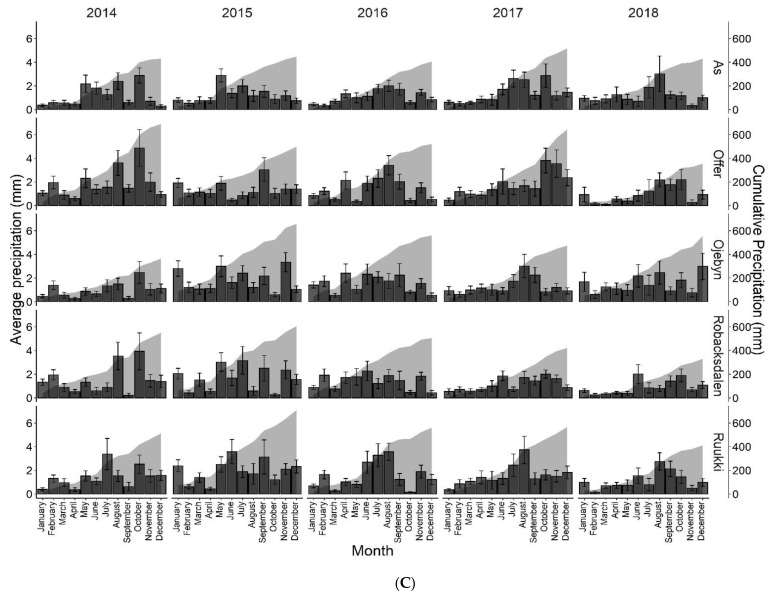
Weather data from 2014 to 2018 for the studied locations in northern Sweden and Finland. (**A**) Black points represent global radiation and the red line represents the radiation for clear sky. (**B**) The black circles represent maximum temperature and olive green circles represent minimum temperature. (**C**) The bars represent the daily average precipitation for each month, and error bars are standard errors. Area graphs are cumulated precipitation from January to December with the magnitudes on the right-hand *y*-axis.

Daily weather data for Swedish locations were obtained from the online database of the Swedish Meteorological and Hydrological Institute (www.smhi.se, access date: 28 November 2019 for Röbäcksdalen; 29 November 2019 for Öjebyn; 22 October 2019 for Offer; 10 Spetember 2019 for Ås) and the Swedish University of Agricultural Sciences (www.ffe.slu.se/lm/LMHome, access date: 28 November 2019 for Röbäcksdalen; 29 November 2019 for Öjebyn; 22 October 2019 for Offer; 10 Spetember 2019 for Ås). For Finland, the data were obtained from the Finnish Meteorological Institute (https://en.ilmatieteenlaitos.fi/, access date: 20 November 2019). The preference was to obtain measured station data. However, for missing days, the data were obtained from the gridded system from the same sources. The weather factors affecting phenological development are presented in Figure 5.

### 4.5. Description of Phenology Modules of APSIM 7.9 and APSIM-NG

The term “APSIM Classic” is used to refer to APSIM7.x versions; in this study, it specifically refers to APSIM 7.9. APSIM Classic simulates barley phenology using a thermal time approach derived from [66,67], with the temperature response characterized by a base, optimum, and maximum, with linear relationships between the critical temperatures. The thermal time target between emergence and floral initiation is adjusted by day length [68,69]. The thermal time from emergence to floral initiation, divided by the plastochron, determines the total leaf number. Vernalisation affects phenology from emergence to floral initiation by regulating the duration of thermal time with a vernalization factor. The vernalization factor is computed from daily maximum and minimum temperature, which, based on the temperature requirements for vernalization, can prolong the vernalization sensitive phase until the requirements are met. Water and nitrogen stresses affect leaf appearance rate, which, depending on intensity, can delay phenology during the vegetative stages. The duration of the developmental stages from anthesis to end of grain filling is solely simulated through thermal time targets. The mechanism was implemented in most of the APSIM 7.x cereal models, including the spring barley model, by adapting the APSIM-Wheat module. A detailed description of the APSIM-Wheat module with the parameters and factors affecting phenology is available online (www.apsim.info, access date: 25 February 2020). Differences between APSIM-Wheat and APSIM-spring barley models are reported by [70].

As the plant modelling framework was imported from APSIM Classic [71] to develop the APSIM-NG models [72], many processes of the growth and development of the APSIM-Barley NG model are the same. The major difference arises in APSIM-NG’s use of Kirby’s Framework [42,43] to capture the effects of vernalisation and photoperiod. According to the framework, the simulation of the timing of the anthesis is dependent on the timing of the flag leaf appearance. It assumes that flag leaf appearance is dependent on the Final Leaf Number, which sets a target, and leaf appearance rate (phyllochron), which sets the rate to proceed toward the target to accumulate thermal time. The accumulation of thermal time and a cultivar specific phyllochron, which changes with the Haun stage, regulate the Leaf appearance rate [73]. The Haun system refers to the leaf development stage by expressing leaf length for each emerging leaf during the crop developmental cycle [74]. The requirements of low temperature for vernalisation and day length for photoperiod decrease with successive Haun stages as the temperature and day length increase and the crop proceeds towards anthesis. The APSIM-NG approach assumes more synchronicity and continuity among the processes, such as leaf emergence, floral primordia initiation, and anthesis, as they are simulated in parallel and are dependent on each other. However, APSIM 7.x simulates phenology with a target-based thermal time approach, independent of leaf appearance, and other processes related to anthesis, and hence there is less synchronicity and continuity among the processes. A detailed description of modelling the final leaf number, different developmental stages, and the effect of photoperiod and vernalisation in APSIM-NG, is available online (https://apsimnextgeneration.netlify.app/, access date: 25 February 2020).

Brown et al. [71] recently outlined the modernisation approaches for the advancements of interface and execution in APSIM-NG with APSIM “Classic”. Brown et al. [72] described the development of crop models in APSIM-next generation from the software, interface and the usage of larger datasets as a suggestion for the advancement of the modern crop models using the wheat module as a case study. The generic processes and functions in the APSIM-NG barley module are the same as the wheat module; however, they are not yet published [72].

### 4.6. Model Ccalibration: Using APSIM’s In-Built Factorial-Based Approach

The models were investigated in two steps: (1) calibration: variety-specific parameters regulating the phenology were calibrated using one dataset, and (2) evaluation: the model performance with the calibrated parameters was statistically evaluated using another dataset. For calibration, a factorial based approach applying an in-built system to create factorial combinations of pre-determined parameter value ranges in the Windows-based modelling software of APSIM-NG was performed. For APSIM 7.9, the same approach was implemented, using R programming with the *apsimr* package. Recently, this calibration approach was applied to calibrate the APSIM-NG maize model [32]. After executing the calibration set-ups, the best set of parameter values was extracted based on minimizing root mean square error (RMSE).

The calibration for each variety was performed with a large number of combinations of phenology-related parameter values (see Table 5). Descriptions of the parameters regulating specific growth stages are provided in Table S2. The selected phenology parameters and their ranges for creating the combinations were chosen based on a manual sensitivity analysis changing one parameter at a time, values of the default cultivars in APSIM, and expert knowledge of the crop phenology in the region. The values of parameters that were not calibrated were kept as in the base cultivars in the APSIM systems. The parameter *tt_emergence,* which regulates days to emergence after sowing in APSIM 7.9, was kept constant, and the value was determined based on the observations recorded at Röbäckdalen during 2017 and 2018. The total number of combinations of the parameters was 14,560 for APSIM 7.9 and 21,600 for APSIM-NG. The numbers for the two models were different because the calibration values of some parameters were higher in APSIM-NG. The simulations with all the parameter combinations were performed for all studied years and locations, as shown in Table 6. RMSEs of the estimated days to anthesis and physiological maturity were calculated using the R programming language. All the codes are provided in Supplementary Material (Codes in R programming).

Three independent calibrations were performed; calibration of days to anthesis (*Calibration 1_AN*) and two calibrations for days to physiological maturity (*Calibration 2_PM* and *Calibration 3_PM*). The dataset for these calibrations is presented in Table 6. *Calibration 1_AN* was for parameters that regulate days to anthesis, i.e., *tt_end_of_juvenile, tt_floral_initiation, vern_sens*, and *photop_sens* for APSIM 7.9 and *BasePhyllo, MinimumLeafNumber, VrnSensitivity, PpSensitivity*, and *EarlyReproductivePpSensitivity* for APSIM-NG (Table 5). *Calibration 2_PM* and *Calibration 3_PM* covered all the parameters that regulate days to anthesis plus the parameters that regulate days from anthesis to physiological maturity (*tt_start_grain_fill* for APSIM 7.9 and *GrainFill* for APSIM-NG). These parameters regulate thermal time accumulation and aim, directly or indirectly, to simulate different phenological stages. The models respond differently to calibrations using different datasets [75]; in this study, both versions of APSIM were tested with two different data sizes. Therefore, the two calibrations differed in terms of the data available for calibrations: *Calibration 2_PM* with fewer data and *Calibration 3_PM* with more data. A total of 20 combinations of locations and seasons generated 291,200 and 432,000 independent simulations for APSIM 7.9 and APSIM-NG in *Calibration 3_PM*.

The calibration and evaluation datasets were categorized based on (1) the available data on two growth stages, and (2) temperature variation at the locations. Data on anthesis and physiological maturity at Röbacksdalen for 2017–2018 were used in *Calibration 1_AN* and *Calibration 2_PM*, and the remaining dataset in *Evaluation 2_PM* was for the parameters which were in calibrated in *Calibration 2_PM.* Offer was the warmest location in terms of maximum temperature and Ruukki was the warmest in terms of minimum temperature. Because of this, the years 2015–2016 for Offer and Ruukki were selected in *Evaluation 3_PM* for the parameters calibrated in *Calibration 3_PM* using other locations and years of data. In *Calibration 1_AN*, to obtain a better calibration for anthesis, we used both years of available data, instead of splitting one year for calibration and another for evaluation. Although this approach did not allow us to evaluate the parameters calibrated under *Calibration 1_AN*, we chose this approach because the calibration of the parameters using two years of data is more robust compared with that using a single year of data.

The parameters which were calibrated under *Calibration 1_AN* could not be statistically evaluated, since all two seasons of data were used for the calibration of anthesis. Because of that, the statistical evaluation, using an independent dataset, was performed only for the calibrated parameters under *Calibration 2_PM and Calibration 3_PM,* which were denoted as *Evaluation 2_PM and Evaluation 3_PM,* respectively. For *Evaluation 2_PM,* data were used from 22 environments and for *Evaluation 3_PM,* data were from four different environments. The evaluation datasets and calibration datasets are summarized in Table 6.

### 4.7. Description of the Selection of Best Parameter Combinations

Both models were run with all the factorial combinations of the parameters for two years (2017 and 2018) for the Röbäcksdalen site. The days to anthesis were extracted for all the individual combinations for the two years. Further, the RMSE was computed using the observed data on 12 varieties for the corresponding site and years. In cases when several parameter combinations resulted in RMSE, the single set of parameter combinations was chosen for each variety based on the frequency of individual parameter values among the equally good parameter sets. For a detailed example of the procedure, please see Supplementary Materials.

### 4.8. Statistical Determinants to Assess the Model Calibration and Evaluation

The best sets of parameter combinations, i.e., those simulating a phenology closest to the observed data, were identified by minimizing root mean square error (RMSE) between estimated and observed dates of anthesis and physiological maturity. Model performances with the calibrated parameters were statistically evaluated with independent datasets. RMSE is a commonly used indicator to evaluate models but it does not highlight the source or type of error, which can be important in refining the models. Therefore, the systematic (RMSE_sys_) and non-systematic (RMSE_nos_) error components of RMSE were also computed [76]. RMSE indicates the mean deviation of the predicted values of the variables under evaluation with respect to the observed ones. RMSE_sys_ indicates the systematic error associated with the model’s predictive ability. A higher magnitude of RMSE_sys_ than zero indicates bias in the model performance. RMSE_nos_ indicates unexplained error. Besides the RMSE error terms, the coefficient of determination (*r*^2^) was also computed for the overall relationship of simulation and measured datapoints.

As RMSE and *r*^2^ are the commonly used determinants, only computations of RMSE_sys_ and RMSE_nos_ are provided below

$${RMSE}_{sys}=\sqrt{\frac{1}{n}\sum_{i=1}^{n}{\left(o_{i}-{\hat{s}}_{i}\right)}^{2}}$$

$${RMSE}_{nos}=\sqrt{\frac{1}{n}\sum_{i=1}^{n}{\left(s_{i}-{\hat{s}}_{i}\right)}^{2}}$$

where $o_{i}$ is observed values of the tested variable, $s_{i}$ is simulated values of the tested variable, *n* is the number of entries for $o_{i}$ and $s_{i}$, ${\hat{s}}_{i}=a+bo_{i}$, with a and b as the intercept and the slope of the regression model, respectively.

## 5. Conclusions

The better performance of the phenology algorithm in APSIM-NG in the calibration indicated its potential suitability at high latitudes compared to the algorithm in APSIM 7.9. However, in the evaluation APSIM-NG showed a greater tendency to overestimate days to physiological maturity. The combination of the best parameters simulating different growth stages close to the observed data changed with the calibrations and size of the datasets used for the calibration. The differences in both algorithms are potentially associated with the methods used to compute thermal time, using different cardinal temperatures. To further improve the performance and suitability of APSIM-NG at high latitudes, we suggest reviewing the cardinal temperatures in the current algorithm and reviewing the elimination of crown temperature in thermal time computation. Hence, more research is suggested on the conception of thermal time computations and their implementation in APSIM-NG.

## Figures and Tables

**Table 1:** Phenology data for twelve spring barley varieties for 2014–2018 cropping seasons with the sowing dates at the studied locations. AN: days to anthesis after sowing; PM: days to physiological maturity after sowing; 6R and 2R refer to 6- and 2-row barley varieties.

Varieties	Röbäcksdalen							Öjebyn					Offer					Ås				Ruukki				
	2014	2015	2016	2017		2018		2014	2015	2016	2017	2018	2014	2015	2016	2017	2018	2014	2015	2017	2018	2014	2015	2016	2017	2018
	PM	PM	PM	AN	PM	AN	PM	PM	PM	PM	PM	PM	PM	PM	PM	PM	PM	PM	PM	PM	PM	PM	PM	PM	PM	PM
Alvari (6R)				59	116	53	84				110	85				99	75			131		84				
Anneli (2R)			100	59	116	54	87			104	108	87			107	103	77			136	83					
Aukusti (6R)	84	105	87	54	109	51	84	74	100	99	105	81	71	100	89	98	74	81	92	125		78	100	87	104	78
GN10063 (6R)			98	52	122	51	84			100	108	79				103	74			141						
Judit (6R)	76	108	93	54	108	51	83	73	98	97	105	78	70	98	89	93	74	80	83	126						
Kaarle (6R)		117	100	59	130	54	87		112	126	112			109	116	109			105	146						80
Kannas (2R)	88	111	105	57	124	54	84	84	110	107	108	86	79	108		104	75	97	98	142	83					
Rödhette (6R)				59	132	54	89				116	92				109	77			144	82					
Severi (6R)	81	116	91	59	118	51	87	76	106	102	109	80	72	106	95	102	74	88	97	130						
Vertti (6R)		98	91	52	117	51	81		103	91	107			101	84	94			88	124		78				
Vilde (6R)	83	103	90	57	120	54	85	75	104	97	108	82	72	103	88	101	75	86	96	126		80	101	89	104	
Vilgott (2R)	96	121	104	59	118	55	88	87	114	112	112	90	81	112	99	110	77	103	109	139	89					
Mean	84	110	96	57	119	53	85	78	106	104	109	84	74	105	96	102	75	89	96	134	84	80	101	88	104	79
SD	7	8	6	3	7	2	2	6	6	10	3	5	5	5	11	6	1	9	9	8	3	3	1	1	0	1
Sowing date	28 May	9 Jun	2 Jun	3 Jun		23 May		23 May	18 Jun	7 Jun	10 Jun	12 Jun	28 May	27 May	30 May	1 Jun	19 May	30 May	28 May	25 May	20 May	20 May	22 May	9 May	30 May	16 May

Agronomic practices: Fertiliser application, recommended; seed rate, 500 seeds m^−2^; Plot size, 1.5 × 9 m; Number of rows/plots, 12; row spacing, 12.5 cm.

**Table 2:** Calibrated parameters for APSIM 7.9. *Calibration 1_ AN*, *Calibration 2_ PM*, and *Calibration 3_PM* represent calibration for days to anthesis, days to physiological maturity, and days to physiological maturity using data from 2, 2, and 22 environments, respectively. ^o^*Cd* and RMSE are degree days and root mean square error, respectively.

Variety												
Parameter	Alvari	Anneli	Aukusti	GN10063	Judit	Kaarle	Kannas	Rodhette	Severi	Vertti	Vilde	Vilgot
*Calibration 1_ AN*												
photop_sens	1	1	0	0	0	1	0	1	1	0	0	0
vern_sens	0	0	1	0.5	1	0	0	0	0	0.5	0	0.5
tt_end_of_juvenile (^o^Cd)	300	300	200	200	200	300	300	300	300	200	300	300
tt_floral_initiation (^o^Cd)	320	320	300	320	300	320	320	320	300	320	320	300
tt_start_grain_fill (^o^Cd)												
RMSE (d)	1.3	0.7	0	1	0.4	0.7	0	0.7	2	0.8	0	0
*Calibration 2_PM*												
photop_sens	6	1	0	3	1	3	1	6	0	6	0	0
vern_sens	0	0	0.5	0	0	0	0	0	0.5	0	0.5	0.5
tt_end_of_juvenile (^o^Cd)	250	300	200	200	200	350	300	300	250	250	300	250
tt_floral_initiation (^o^Cd)	300	320	300	320	300	300	320	320	300	300	320	300
tt_start_grain_fill (^o^Cd)	525	525	575	625	575	550	575	550	575	525	500	575
RMSE (d)	4.5	3	2.2	7.5	2.5	8.6	8.2	8.5	4	6.7	6	3.5
*Calibration 3_PM*												
photop_sens	1	1	3	3	1	3	1	3	0	1	1	3
vern_sens	0	0	0	0	0	0	0	0	0	0	0	0
tt_end_of_juvenile (^o^Cd)	450	400	250	450	400	450	400	450	450	450	450	450
tt_floral_initiation (^o^Cd)	260	320	300	240	280	240	320	240	240	240	240	260
tt_start_grain_fill (^o^Cd)	400	400	450	400	350	450	400	450	400	350	350	400
RMSE (d)	8.3	7.8	7.5	10.4	8.7	11	10.1	11	9.9	8.7	8.5	7.6

**Table 3:** Model evaluation determinants. *Evaluation 2_PM* represents statistical evaluation of the parameter calibrated under *Calibration 2_ PM*; *Evaluation 3_PM* represents statistical evaluation of the parameter calibrated under *Calibration 3_ PM*. Intercept, Slope, RMSE, RMSE_sys_, RMSE_nos_, and *r*^2^ represent regression intercept and slope, root mean square error, systematic component of RMSE, non-systematic component of RMSE, and coefficient of determination, respectively.

Intercept	APSIM 7.9		APSIM-NG	
	Evaluation 2_PM *	*Evaluation 3_PM*	*Evaluation 2_PM ^¤^*	*Evaluation 3_PM*
	−32.7	−2.2	2.3	18
Slope	1.2	0.99	0.87	0.81
RMSE	11.4	4.73	13.6	5.8
RMSE_sys_	10.17	3.1	10.19	2
RMSE_nos_	20.64	7.15	22.63	5.81
*r* ^2^	0.74	0.81	0.78	0.63

* Based on complete 159 pairs of simulated and observed datapoints (Figure 2A). ^¤^ Based on complete 139 pairs of simulated and observed datapoints. 20 pairs that did not reach physiological maturity were excluded (Figure 3A).

**Table 4:** Calibrated parameters for APSIM Next Generation (APSIM-NG). *Calibration 1_ AN, Calibration 2_ PM, and Calibration 3_ PM* represent calibration for days to anthesis, days to physiological maturity, and days to physiological maturity using data from 2, 2, and 22 environments, respectively.

Variety												
Parameter	Alvari	Anneli	Aukusti	GN10063	Judit	Kaarle	Kannas	Rodhette	Severi	Vertti	Vilde	Vilgot
*Calibration 1_AN*												
*PpSensitivity*	0	0	0	0	0	0	0	0	0	0	0	0
*VrnSensitivity*	1.5	1.5	0.5	0.5	1	1.5	1.5	1.5	0.5	0.5	1.5	1.5
*BasePhyllo (* ^o^ *Cd)*	60	60	60	60	55	60	60	60	60	55	60	60
*MinimumLeafNumber*	6	6	6	6	6	6	6	6	6	6	6	6
*GrainFill (* ^o^ *Cd)*												
*EarlyReproductivePpSensitivity*	0	0	0	0	0	0	0	0	0	0	0	0
*RMSE* (d)	0	0	0	0	0	0	0	0	0	0	0	0
*Calibration 2_PM*												
*PpSensitivity*	0	0	0	0	0	0	0	0	0	0	0	0
*VrnSensitivity*	1.5	1.5	1.5	1	1	1	0.5	0.5	1.5	1.5	0.5	1.5
*BasePhyllo (* ^o^ *Cd)*	50	55	50	50	60	50	50	50	50	50	50	60
*MinimumLeafNumber*	5	5	6.5	5	5	5	5	5	6	6	5	5
*GrainFill (* ^o^ *Cd)*	525	525	450	550	450	600	600	625	500	500	575	500
*EarlyReproductivePpSensitivity*	0	0	0	0	0	0	0	0	0	0	0	0
*RMSE* (d)	0	0	0	0	0	0	0	0	0	0	0	0
*Calibration 3_PM*												
*PpSensitivity*	0	0	0	0	0	0	0	0	0	0	0	0
*VrnSensitivity*	0.5	0	0	0	0.5	3	0.5	0.5	3	0.5	0.5	0
*BasePhyllo (* ^o^ *Cd)*	65	60	50	65	65	55	60	50	55	60	60	50
*MinimumLeafNumber*	5	6	6.5	6.5	5	5	5	5	5	5	5	6.5
*GrainFill (* ^o^ *Cd)*	450	450	450	400	400	450	500	575	400	450	450	525
*EarlyReproductivePpSensitivity*	0	0	0	0	0	0	0	0	0	0	0	0
*RMSE* (d)	4.6	4.1	5.5	6.2	7.0	6.6	7.0	7.5	7.2	6.8	6.7	4.9

**Table 5:** Parameters and their levels applied in factorial calibration of APSIM 7.9 and APSIM-NG barley phenology models. The total number of combinations for APSIM 7.9 and APSIM-NG were 14,560 and 21,600, respectively.

Factorial-Based Calibration					
APSIM 7.9			APSIM-NG		
Parameter	Default Value	Level	Parameter	Default Value	Level
tt_emergence (°Cd)	1	40	BasePhyllo (^o^Cd) *	50	50, 55, 60, 65, 70, 75
tt_end_of_juvenile (°Cd) *	400	150, 200, 250, 300, 350, 400, 450	MinimumLeafNumber *	9	5, 5.5, 6, 6.5, 7
tt_floral_initiation (°Cd) *	230	180, 200, 220, 240, 260, 280, 300, 320	GrainFill (^o^Cd)¤	540	350, 400, 450, 500, 525, 550, 575, 600, 625, 650, 675, 700
tt_start_grain_fill (°Cd)¤	545	350, 400, 450, 500, 525, 550, 575, 600, 625, 650, 675, 700, 750	VrnSesnsitivity*	0	0, 0.5, 1, 1.5, 3
vern_sens *	1.5	0, 0.5, 1, 1.5, 3	PpSesnsitivity *	3	0, 1, 3, 6
photop_sens *	3	0, 1, 3, 6	EarlyReproductivePpSesnsitivity *	0	0, 1, 2

* Regulating days to anthesis. ^¤^ Regulating days after anthesis to physiological maturity.

**Table 6:** Calibration and evaluation datasets for APSIM 7.9 and APSIM-NG spring barley models. *Calibration 1_AN* represents calibration of days to anthesis and *Calibration 2_PM,* and *Calibration 3_PM* represent calibration of days to physiological maturity. *Evaluation 2_PM* and *Evaluation 3_PM* represent the independent evaluation of *Calibration 2_PM,* and *Calibration 3_PM,* respectively.

Growth Stage	Location	Calibration Dataset			Evaluation Dataset		
		*Calibration 1_AN*	*Calibration 2_PM*	*Calibration 3_PM*		*Evaluation 2_PM*	*Evaluation 3_PM*
		Season	Season	Season	Location	Season	Season
Days to anthesis	Röbäcksdalen	2017–2018					
Days to physiological maturity	Röbäcksdalen		2017–2018		Röbäcksdalen	2014–2016	
Days to physiological maturity	Röbäcksdalen			2014–2018			
	Ås			2014, 2016–2018	Ås	2014, 2016–2018	
	Öjebyn			2014–2018	Öjebyn	2014–2018	
	Offer			2014, 2017–2018	Offer	2014–2018	2015–2016
	Ruukki			2014, 2017–2018	Ruukki	2014–2018	2015–2016

## Supplementary Materials

The following are available online at https://www.mdpi.com/2223-7747/10/3/443/s1. Codes in R programming, Table S1: Soil water characteristics at Röbäcksdalen, Öjebyn, Offer, Ås and Ruukki used to run APSIM 7.9 and APSIM-NG barley models. The bolded figures were assumed to complete the entries for the soil profile up to 1 m, Table S2: Description of the parameters used for the factorial combinations for calibration of APSIM 7.9 and APSIM-NG barley phenology models, Table S3: Combinations of the parameters resulted in the same lowest RMSE for Alvari and Anneli. The highlighted parameter combinations for the varieties were selected based on the frequency of individual parameters (Figure S2), which are presented in Table 5, Figure S1: Frequency of RMSE with the combinations of all calibrated parameters and varieties for *Calibration 1_AN* and RMSEs of Alvari and Anneli with the respective legend, RMSE_Alvari and RMSE_Anneli, Figure S2: Frequency of selection of individual parameter values in APSIM 7.9 resulted in the lowest RMSE for the varieties.
