# Supplementary material for: Comparative Analysis of Phenology Algorithms of the Spring Barley Model in APSIM 7.9 and APSIM Next Generation: A Case Study for High Latitudes
Source: Plants (Basel). 2021 Feb 26;10(3):443. doi: 10.3390/plants10030443 (PMC7996762; doi:10.3390/plants10030443)
Supplement: Supplementary file 1 [file plants-10-00443-s001.zip › plants-1107907-supplementary/Supplimentary Materials (tables and figures).docx]

**Supplementary Materials: Comparative Analysis of Phenology Algorithms of the Spring Barley Model in APSIM 7.9 and APSIM Next Generation: A Case Study for High Latitudes**

**Uttam Kumar, Julien Morel, Göran Bergkvist, Taru Palosuo, Anne-Maj Gustavsson, Allan Peake, Hamish Brown, Mukhtar Ahmed and David Parsons**

**Table S1.** Soil water characteristics at Röbäcksdalen, Öjebyn, Offer, Ås and Ruukki used to run APSIM 7.9 and APSIM-NG barley models. The bolded figures were assumed to complete the entries for the soil profile up to 1 m.

| **Depth (cm)** | **Saturation Point (g/g)** | **Field Capacity**  **(pF 1 m) (g/g)** | **Wilting Point (g/g)** | **Dry bulk Density (g/cmᶾ)** | **pH** |
| --- | --- | --- | --- | --- | --- |
| Röbacksdalen | | | | | |
| 0-10 | 37.72 | 34.20 | 6.43 | 1.29 | 5.72 |
| 10-20 | 46.75 | 35.32 | 6.44 | 1.01 | 5.47 |
| 20-40 | 62.21 | 44.78 | 6.61 | 0.88 | 5.31 |
| 20-40 | 62.21 | 44.78 | 6.61 | 0.88 | 5.31 |
| 40-60 | 50.91 | 42.74 | 6.76 | 1.10 | 4.72 |
| 40-60 | 50.91 | 42.74 | 6.76 | 1.10 | 4.72 |
| 60-90 | 40.65 | 38.20 | 6.16 | 1.30 | 4.21 |
| 60-90 | 40.65 | 38.20 | 6.16 | 1.30 | 4.21 |
| 60-90 | 40.65 | 38.20 | 6.16 | 1.30 | 4.21 |
| 90-120 | 44.25 | 41.29 | 5.81 | 1.22 | 3.87 |
| Öjebyn | | | | | |
| 0-10 | 45.55 | 37.60 | 14.35 | 1.28 | **5.72** |
| 10-20 | 45.55 | 32.25 | 14.35 | 1.28 | **5.47** |
| 20-40 | 50.05 | 32.75 | 15.10 | 1.42 | **5.31** |
| 20-40 | 44.25 | 31.25 | 18.60 | 1.44 | **5.31** |
| 40-60 | 44.00 | 31.40 | 20.15 | 1.43 | **4.72** |
| 40-60 | 42.50 | 36.55 | 6.40 | 1.51 | **4.72** |
| 60-90 | 41.90 | 31.75 | 2.55 | 1.53 | **4.21** |
| 60-90 | 46.90 | 31.65 | 12.20 | 1.41 | **4.21** |
| 60-90 | 47.15 | 36.00 | 16.80 | 1.40 | **4.21** |
| 90-120 | 43.90 | 40.15 | 3.65 | 1.53 | **3.87** |
| Offer | | | | | |
| 0-10 | 33.95 | 29.54 | 6.28 | 1.36 | 6.16 |
| 10-20 | 36.04 | 29.87 | 6.23 | 1.30 | 6.2 |
| 20-40 | 30.50 | 27.85 | 6.55 | 1.45 | 6.3 |
| 20-40 | 30.50 | 27.85 | 6.55 | 1.45 | 6.3 |
| 40-60 | 30.19 | 26.99 | 6.26 | 1.38 | 6.54 |
| 40-60 | 30.19 | 26.99 | 6.26 | 1.38 | 6.54 |
| 60-90 | 30.32 | 28.02 | 6.32 | 1.43 | 6.63 |
| 60-90 | 30.32 | 28.02 | 6.32 | 1.43 | 6.63 |
| 60-90 | 30.32 | 28.02 | 6.32 | 1.43 | 6.63 |
| 90-120 | 31.42 | 29.43 | 6.32 | 1.45 | 6.53 |
| Ås | | | | | |
| 0-10 | 32.71 | 27.53 | 11.14 | 1.35 | 6.11 |
| 10-20 | 33.09 | 21.18 | 10.96 | 1.29 | 6.19 |
| 20-40 | 36.93 | 20.39 | 9.35 | 1.24 | 6.35 |
| 20-40 | 36.93 | 20.39 | 9.35 | 1.24 | 6.35 |
| 40-60 | 19.05 | 15.99 | 11.29 | 1.75 | 6.67 |
| 40-60 | 19.05 | 15.99 | 11.29 | 1.75 | 6.67 |
| 60-90 | 25.62 | 16.96 | 13.74 | 1.34 | 7.18 |
| **60-90** | **25.62** | **16.96** | **13.74** | **1.34** | **7.18** |
| **60-90** | **25.62** | **16.96** | **13.74** | **1.34** | **7.18** |
| **90-120** | **25.62** | **16.96** | **13.74** | **1.34** | **7.18** |
| Ruukki | | | | | |
| **0-10** | **48.00** | **42.30** | **23.30** | **1.32** | **5.72** |
| **10-20** | **48.00** | **42.30** | **23.30** | **1.32** | **5.47** |
| 20-40 | 48.00 | 42.30 | 23.30 | 1.32 | **5.31** |
| **20-40** | **45.00** | **34.00** | **14.60** | **1.50** | **5.31** |
| 40-60 | 45.00 | 34.00 | 14.60 | 1.50 | **4.72** |
| **40-60** | **45.00** | **34.00** | **14.60** | **1.50** | **4.72** |
| **60-90** | **45.00** | **34.00** | **14.60** | **1.50** | **4.21** |
| **60-90** | **45.00** | **34.00** | **14.60** | **1.50** | **4.21** |
| **60-90** | **45.00** | **34.00** | **14.60** | **1.50** | **4.21** |
| **90-120** | **45.00** | **34.00** | **14.60** | **1.50** | **3.87** |

**Table S2.** Description of the parameters used for the factorial combinations for calibration of APSIM 7.9 and APSIM-NG barley phenology models.

| **APSIM 7.9** | | **APSIM-NG** | |
| --- | --- | --- | --- |
| **Parameter** | **Description** | **Parameter** | **Description** |
| tt_emergence (^o^Cd) | Regulates the duration from germination to emergence | BasePhyllo (^o^Cd)* | Regulates rate of leaf appearance |
| tt_end_of_juvenile (^o^Cd)* | Regulates the duration from emergence to beginning of panicle initiation | MinimumLeafNumber * | Regulates the number of leaves |
| tt_floral_initiation (^o^Cd)* | Regulates the duration from the beginning of panicle initiation to the end of the flowering process | GrainFill (^o^Cd)¤ | Regulates the duration from the start of grain filling to the end of grain filling |
| tt_start_grain_fill (^o^Cd)¤ | Regulates the duration from the start of grain filling to the end of grain filling | VrnSesnsitivity* | Regulates vernalisation sensitivity |
| vern_sens * | Regulates vernalisation sensitivity | PpSesnsitivity* | Regulates photoperiod sensitivity |
| photop_sens* | Regulates photoperiod sensitivity | EarlyReproductivePpSesnsitivity* | Regulates the duration for the plant to go from flag leaf ligule appearance at 16 and 8 hours daylength |

Description of the selection of best parameter combinations: a detailed version than in the main paper

The number of combinations of the parameters was 14,560 for APSIM 7.9 and 21,600 for APSIM-NG after combining all the parameter levels mentioned in Table S2. The best set of parameter values was extracted based on minimizing root mean square error (RMSE). *Calibration 1_AN* in particular, resulted in the same lowest RMSEs with different combinations of the parameters (see Table S4) for each variety. Below we describe the process on how the best set of parameter combinations were extracted for the varieties which are reported in Table 2 and 3, for APSIM 7.9 and ASPIM-NG, respectively. This description is only for APSIM 7.9, however, the same process was followed for APSIM-NG.

The factorial combinations were simulated for two years (2017 and 2018) for Röbäcksdalen site with R programming using the *apsimr* package. The days to anthesis were extracted for individual combinations in the factorial file for the two years. Further, the RMSE was computed using the observed data on 12 varieties for the corresponding site and years. Several parameter combinations had the same days to anthesis which, consequently, resulted in the same RMSE. As a result, several sets of parameter combinations had the same lowest (Best) RMSEs (see Table S4). For a visual and quantitative description, the RMSE range and frequency of all combinations (14560 parameters combinations x 2 years x 12 Varieties) are presented in Fig S1A with RMSEs of two example varieties, Alvari and Anneli. The extracted lowest RMSEs of all varieties with Alvari and Anneli are presented in Fig. S1 B. Further, the single set of parameter combinations were extracted for each variety based on the frequency of individual parameter values which are presented in Fig S2. Following the process, the best set of parameter combinations for Alvari and Anneli, as examples, are highlighted in Table S4. A similar process was implemented for all the remaining varieties. The selection of parameters based on frequency corroborates with the fact that they have a large influence on the phenology and hence have a higher chance to simulate days to anthesis more accurately under several environments (years and locations) than others.

**Table S3.** Combinations of the parameters resulted in the same lowest RMSE for Alvari and Anneli. The highlighted parameter combinations for the varieties were selected based on the frequency of individual parameters (Figure S2) which are presented in Table 5.

| **tt_floral_initiation** | **tt_end_of_juvenile** | **photop_sens** | **vern_sens** | **Variety** | **Lowest_RMSE** |
| --- | --- | --- | --- | --- | --- |
| 180 | 400 | 3 | 0 | Alvari | 1.3 |
| 180 | 450 | 0 | 0 | Alvari | 1.3 |
| 220 | 400 | 1 | 0 | Alvari | 1.3 |
| 240 | 350 | 3 | 0 | Alvari | 1.3 |
| 280 | 300 | 0 | 0.5 | Alvari | 1.3 |
| 280 | 300 | 1 | 0.5 | Alvari | 1.3 |
| 280 | 300 | 3 | 0.5 | Alvari | 1.3 |
| 280 | 350 | 0 | 0 | Alvari | 1.3 |
| 300 | 300 | 3 | 0 | Alvari | 1.3 |
| 320 | 300 | 1 | 0 | Alvari | 1.3 |
| 180 | 300 | 0 | 1.5 | Anneli | 0.7 |
| 180 | 300 | 1 | 1.5 | Anneli | 0.7 |
| 180 | 300 | 3 | 1.5 | Anneli | 0.7 |
| 180 | 300 | 6 | 1.5 | Anneli | 0.7 |
| 180 | 350 | 0 | 1 | Anneli | 0.7 |
| 180 | 350 | 1 | 1 | Anneli | 0.7 |
| 180 | 350 | 3 | 1 | Anneli | 0.7 |
| 180 | 350 | 6 | 1 | Anneli | 0.7 |
| 180 | 400 | 3 | 0 | Anneli | 0.7 |
| 180 | 450 | 0 | 0 | Anneli | 0.7 |
| 180 | 450 | 1 | 0 | Anneli | 0.7 |
| 200 | 400 | 3 | 0 | Anneli | 0.7 |
| 200 | 450 | 0 | 0 | Anneli | 0.7 |
| 220 | 400 | 1 | 0 | Anneli | 0.7 |
| 240 | 350 | 0 | 0.5 | Anneli | 0.7 |
| 240 | 350 | 1 | 0.5 | Anneli | 0.7 |
| 240 | 350 | 3 | 0 | Anneli | 0.7 |
| 240 | 350 | 3 | 0.5 | Anneli | 0.7 |
| 240 | 400 | 0 | 0 | Anneli | 0.7 |
| 240 | 400 | 1 | 0 | Anneli | 0.7 |
| 260 | 350 | 3 | 0 | Anneli | 0.7 |
| 280 | 300 | 0 | 0.5 | Anneli | 0.7 |
| 280 | 300 | 1 | 0.5 | Anneli | 0.7 |
| 280 | 300 | 3 | 0.5 | Anneli | 0.7 |
| 280 | 350 | 0 | 0 | Anneli | 0.7 |
| 280 | 350 | 1 | 0 | Anneli | 0.7 |
| 300 | 300 | 0 | 0.5 | Anneli | 0.7 |
| 300 | 300 | 1 | 0.5 | Anneli | 0.7 |
| 300 | 300 | 3 | 0 | Anneli | 0.7 |
| 300 | 300 | 3 | 0.5 | Anneli | 0.7 |
| 300 | 350 | 0 | 0 | Anneli | 0.7 |
| 320 | 200 | 0 | 1.5 | Anneli | 0.7 |
| 320 | 200 | 1 | 1.5 | Anneli | 0.7 |
| 320 | 200 | 3 | 1.5 | Anneli | 0.7 |
| 320 | 200 | 6 | 1.5 | Anneli | 0.7 |
| 320 | 300 | 1 | 0 | Anneli | 0.7 |
| 320 | 300 | 3 | 0 | Anneli | 0.7 |

**
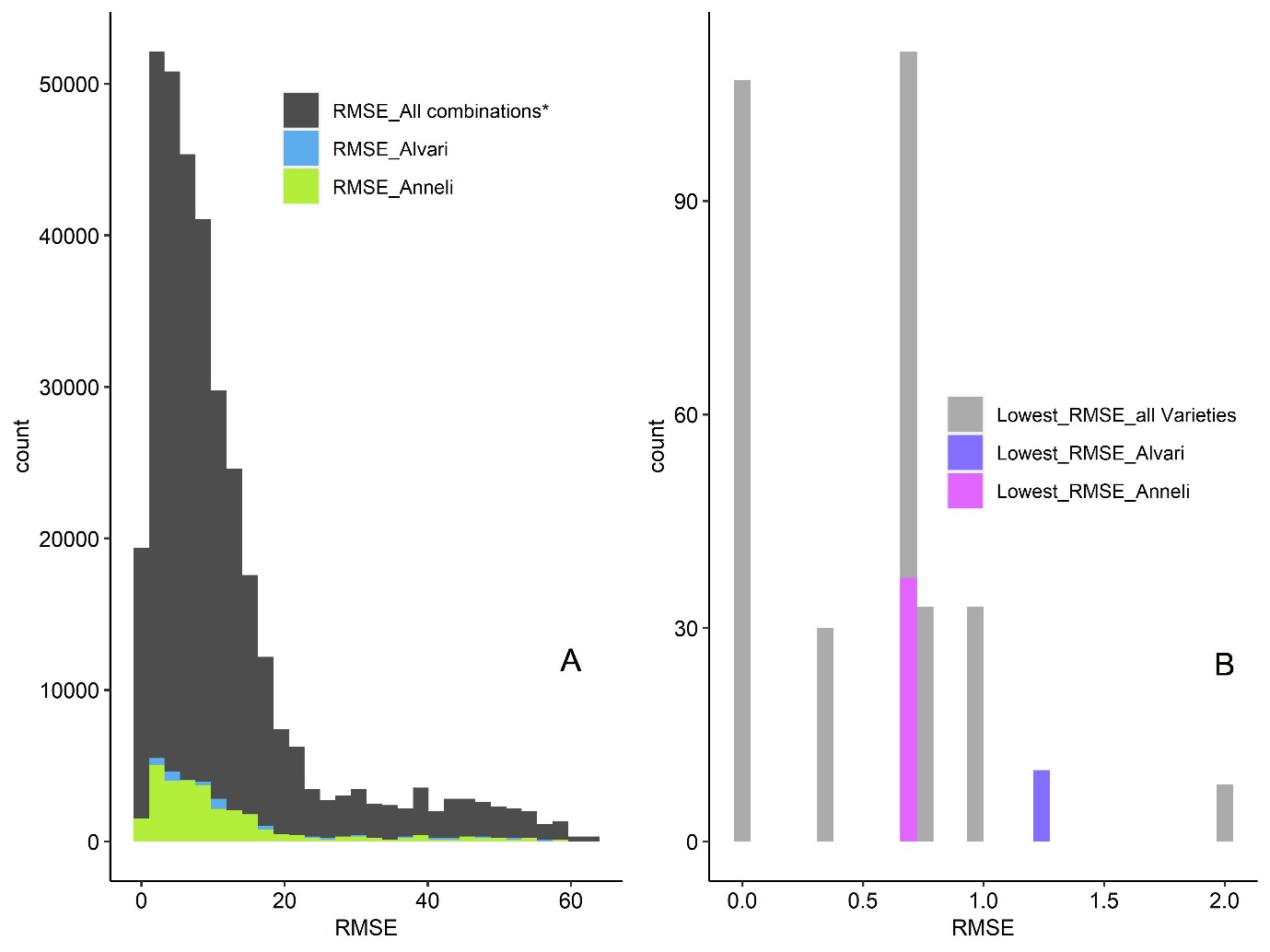
**

**Figure S1.** Frequency of RMSE with the combinations of all calibrated parameters and varieties for *Calibration 1_AN* and RMSEs of Alvari and Anneli with the respective legend, RMSE_Alvari and RMSE_Anneli (**A**). Extracted frequency of the Lowest RMSE for all varieties (Lowest_RMSE_all Varieties), and Alvari (Lowest_RMSE_Alvari) and Anneli (Lowest_RMSE_Anneli), as examples (**B**). Different combinations of the parameters output the same days to anthesis, hence the same RMSE, shown in Table S3 for the example varieties. The frequency of those combinations that resulted in the same RMSE are shown in graph B. * Suggest that RMSE was computed 14,560 parameter combinations × 2 years × 12 varieties.

**
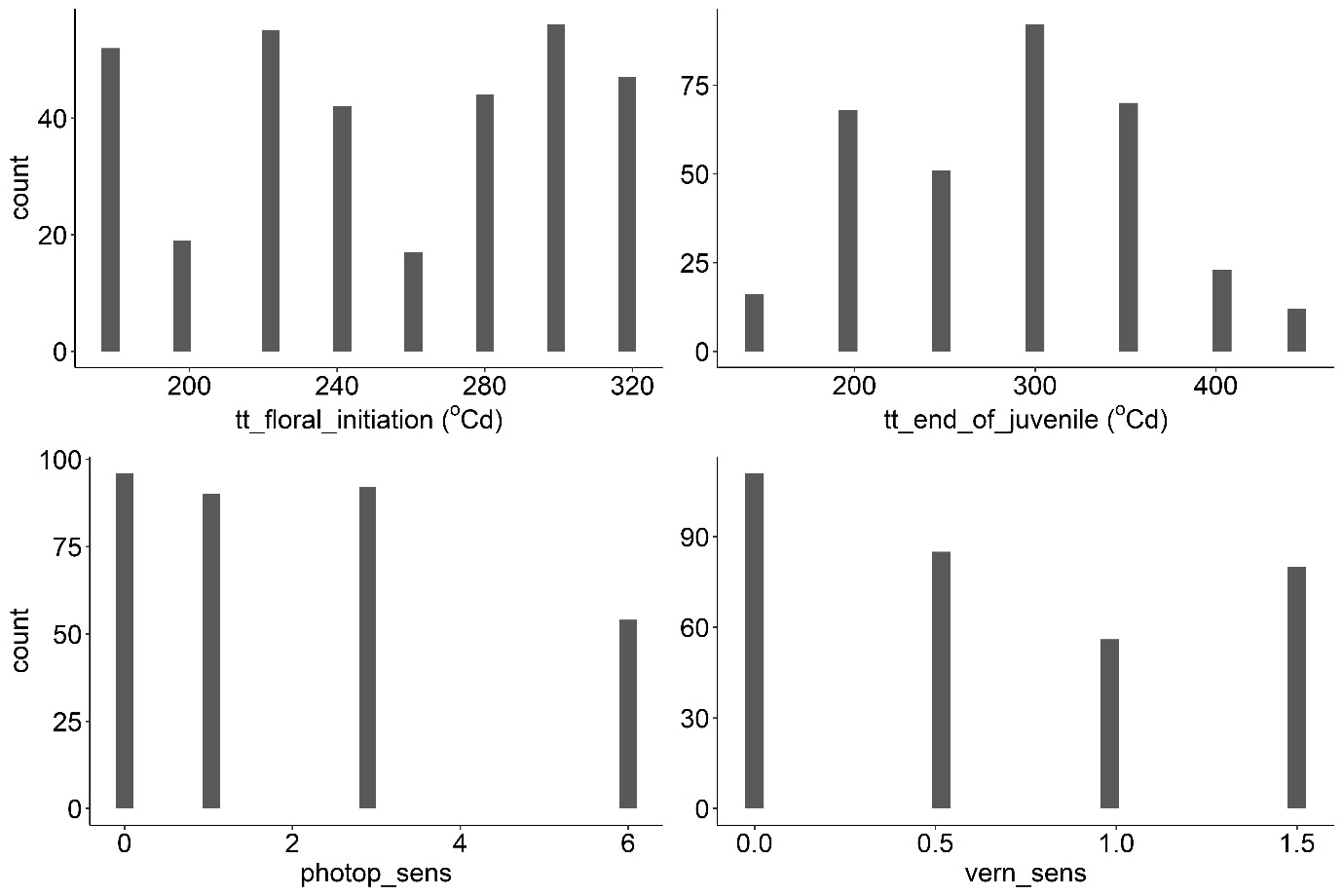
**

**Figure S2.** Frequency of selection of individual parameter values in APSIM 7.9 resulted in the lowest RMSE for the varieties. Based on the highest frequency of individual parameter values, the best combination of parameter values was selected, presented in Table 5.
